# Decompression of a Dentigerous Cyst Treatment in Mixed Dentition: A Case Report with 5 Years Follow-Up

**DOI:** 10.1155/2023/8628326

**Published:** 2023-09-20

**Authors:** Antoine Berberi, Georges Aad, Marise Nassar, Gwenaëlle Maalouf, Nabih Nader

**Affiliations:** ^1^Department of Oral and Maxillofacial Surgery, Faculty of Dental Medicine, Lebanese University, Beirut, Lebanon; ^2^Department of Oral Medicine and Maxillofacial Radiology, Faculty of Dental Medicine, Lebanese University, Beirut, Lebanon

## Abstract

Among developmental odontogenic cysts, the dentigerous type is the second most prevailing one. It is a benign intraosseous lesion commonly affecting the mandibular region. Dentigerous cysts present a high prevalence in children as they can be caused by the eruption of permanent teeth or the infection of deciduous ones. The adopted treatment modalities include enucleation (cystectomy), marsupialization, and decompression. Decompression maintains communication between the cyst and the oral medium through a sutured fixed device, namely an acrylic stent or a pretrimmed disposable suction tube. In the mixed dentition, the extraction of the affected primary teeth and the decompression approach is recommended, especially since children and parents are more tolerant of conservative treatments. We report in this study, a case of a 9-year-old boy complaining of a painful swelling in the left mandibular region. Intraoral and radiological examination revealed an expansion of the buccal and lingual cortical plates associated with teeth #73, #74, and #75 and a well-limited, unilocular radiolucent image extending from the distal aspect of tooth #31 to the mesial aspect of tooth #36 involving the crowns of the unerupted teeth #33, #34, and #35. The preliminary diagnosis was in favor of a dentigerous cyst. The treatment was to extract the deciduous teeth and to use a sterile tube for decompression. The patient was followed up for 5 years, a complete remission of the cyst was observed and the teeth #33, #34, and #35 re-erupted normally on the mandibular arch.

## 1. Introduction

Among developmental odontogenic cysts, the dentigerous type is the second most prevailing one [1]. It is a benign intraosseous lesion commonly affecting the mandibular region. It is causative of teeth dislocation, cortical bone expansion, intraoral swelling, and sometimes facial asymmetry noting that it is usually asymptomatic. Radiographically, this type of cyst shows well-defined sclerotic margins surrounding a unilocular lesion. It can appear diffuse if the cyst is infected [2]. Dentigerous cysts present a high prevalence in children as they can be caused by the eruption of permanent teeth or the infection of deciduous ones [3]. The adopted treatment modalities include enucleation (cystectomy), marsupialization, and decompression. The first consists of complete cyst removal and is indicated for small-sized cysts [4]. With larger lesions, this technique can cause numerous damages hence the consideration of more conservative approaches [2, 5]. Marsupialization is achieved by deroofing the cyst through a large window within the bone and suturing the edges of the incised mucosa to the cystic wall [6]. Decompression maintains communication between the cyst and the oral medium through a sutured fixed device, namely an acrylic stent or a pretrimmed disposable suction tube [7]. In the mixed dentition, the extraction of the affected primary teeth and the decompression approach is recommended, especially since children and parents are more tolerant of conservative treatments [8]. This technique is advantageous as it prevents the surgical damage of developing germs and adjacent anatomical structures with the preservation of oral tissues [9].

We report in this study, a case of dentigerous cyst in the mixed dentition treated with the decompression technique.

## 2. Case Report

**Figure 1:**
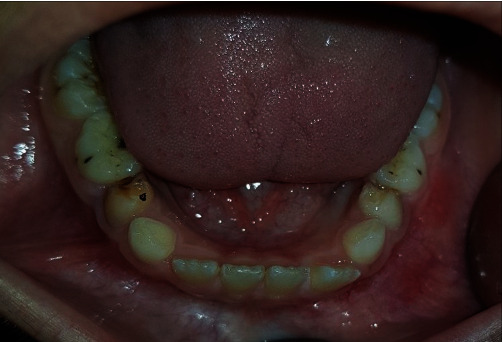
Intraoral view of the mandibular swelling in relation to teeth #73, #74, and #75.

**Figure 2:**
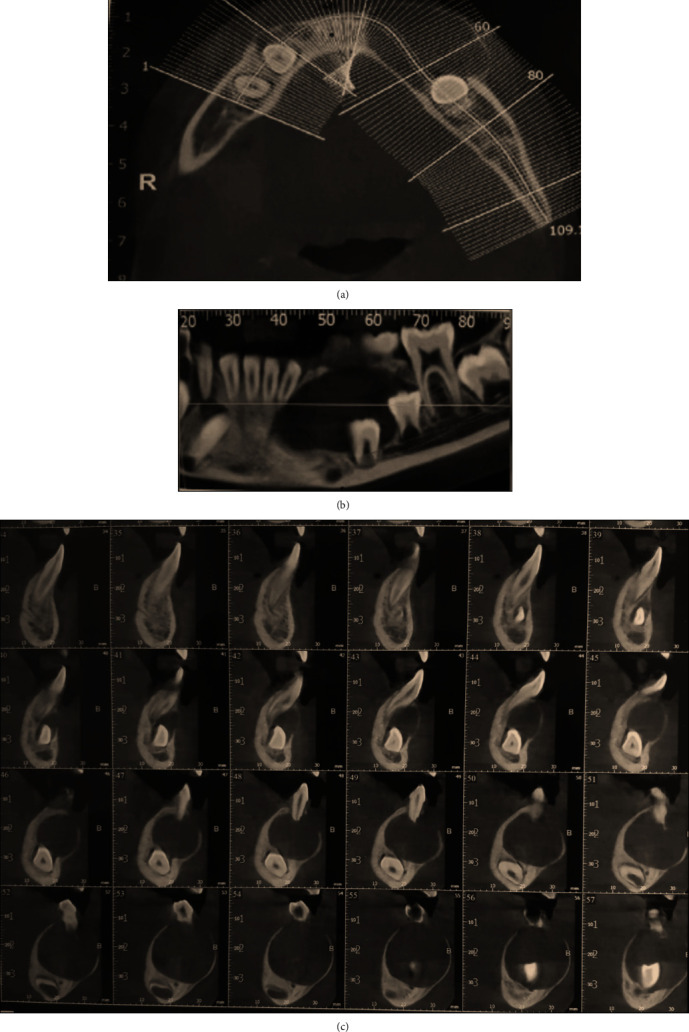
(a) Axial view of the CBCT showing the expanded buccal bone. (b) Panoramic reconstruction revealed a well-limited, unilocular radiolucent image apical to the deciduous teeth with an extension to the mesial of the tooth #36, and the crowns of the unerupted teeth #33, #34, and #35 were pushed towards the basilar mandibular bone. (c) Para-axial cuts showed apical displacement of the teeth buds towards the mental nerve in proximity and an expansion of the buccal and lingual bone plates.

A 9-year-old boy presented to the Department of Oral and Maxillofacial Surgery, complaining of a painful swelling in the left mandibular region. Intraoral examination revealed an expansion of the buccal and lingual cortical plates associated with teeth #73, #74, and #75 (Figure 1). No signs of paresthesia were reported. The vitality test of the premolars was positive. Cone-beam computed tomography (CBCT) was done to evaluate bone involvement in the affected area. The axial cuts displayed an expanded buccal bone (Figure 2(a)) and the panoramic reconstruction revealed a well-limited, unilocular radiolucent image below the apices of teeth #32, #73, #74, and #75. The lesion extended from the distal aspect of tooth #31 to the mesial aspect of tooth #36 involving the crowns of the unerupted teeth #33, #34, and #35 (Figure 2(b)). The para-axial cuts showed apical displacement of the teeth buds towards the mental nerve in proximity to the lower border of the mandible. Expansion of the buccal and lingual bone plates with no perforation was documented as well (Figure 2(c)).

The preliminary diagnosis was in favor of a dentigerous cyst and the differential diagnosis could be a periapical cyst. The treatment plan was in favor of the decompression technique taking into consideration the age of the patient, and the clinical and radiographic findings.

The surgical procedure was explained to and approved by the parents.

**Figure 3:**
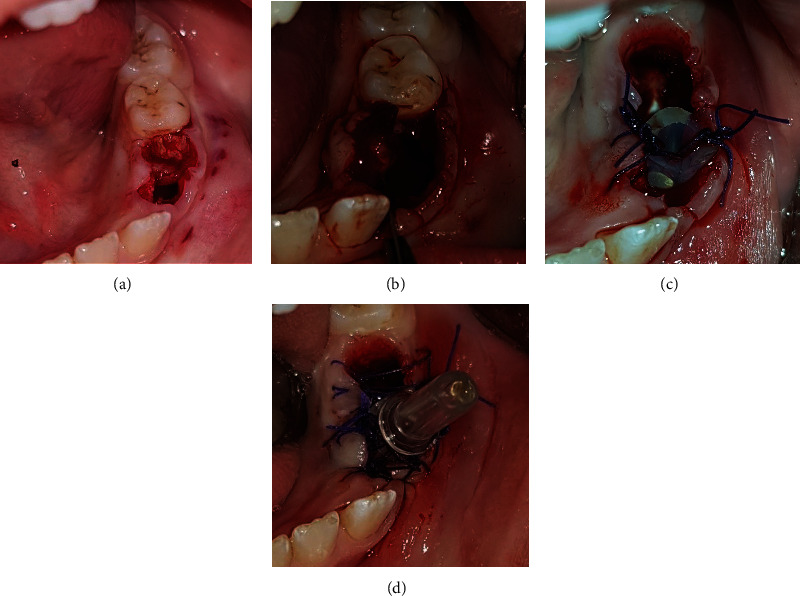
(a) Teeth were extracted. (b) An incisional biopsy of the cystic tissue. (c) A sterile silicon irrigation tube was cut into a small cylinder to serve as a drain. (d) The tube was stabilized in the extracted sockets with multiple interrupted sutures.

**Figure 4:**
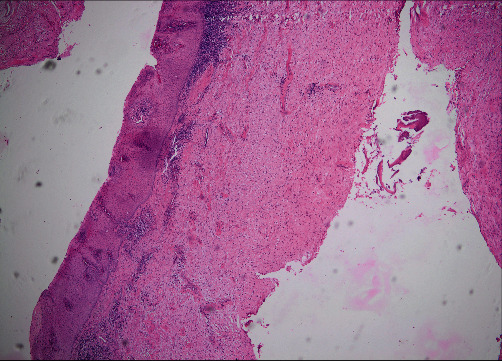
Cystic wall composed of fibrous tissue, lined with a non-keratinized stratified epithelium, H&E ×20.

**Figure 5:**
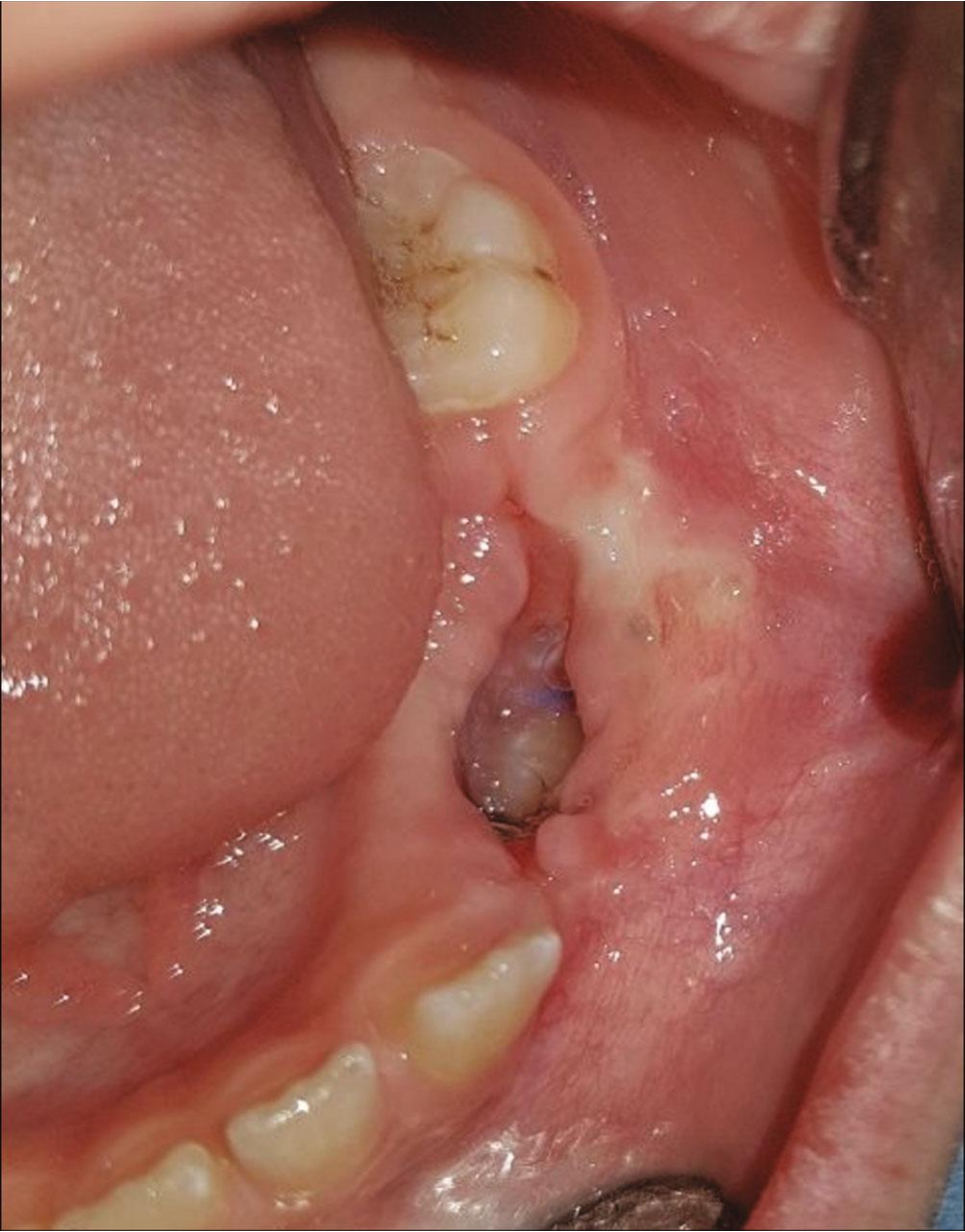
The fixed device used for decompression was removed.

**Figure 6:**
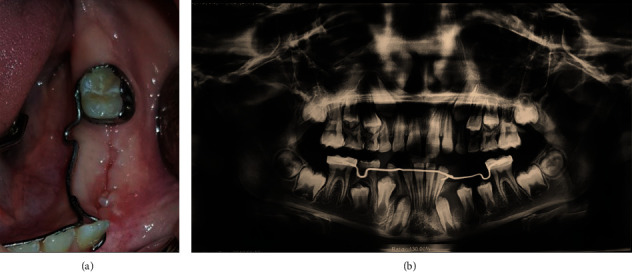
(a) Six months of clinical imaging revealed the eruption of the first permanent premolar. (b) The X-ray showed bone regeneration in the previously cystic region, and the progression of the permanent canine and second premolar teeth buds towards occlusion.

**Figure 7:**
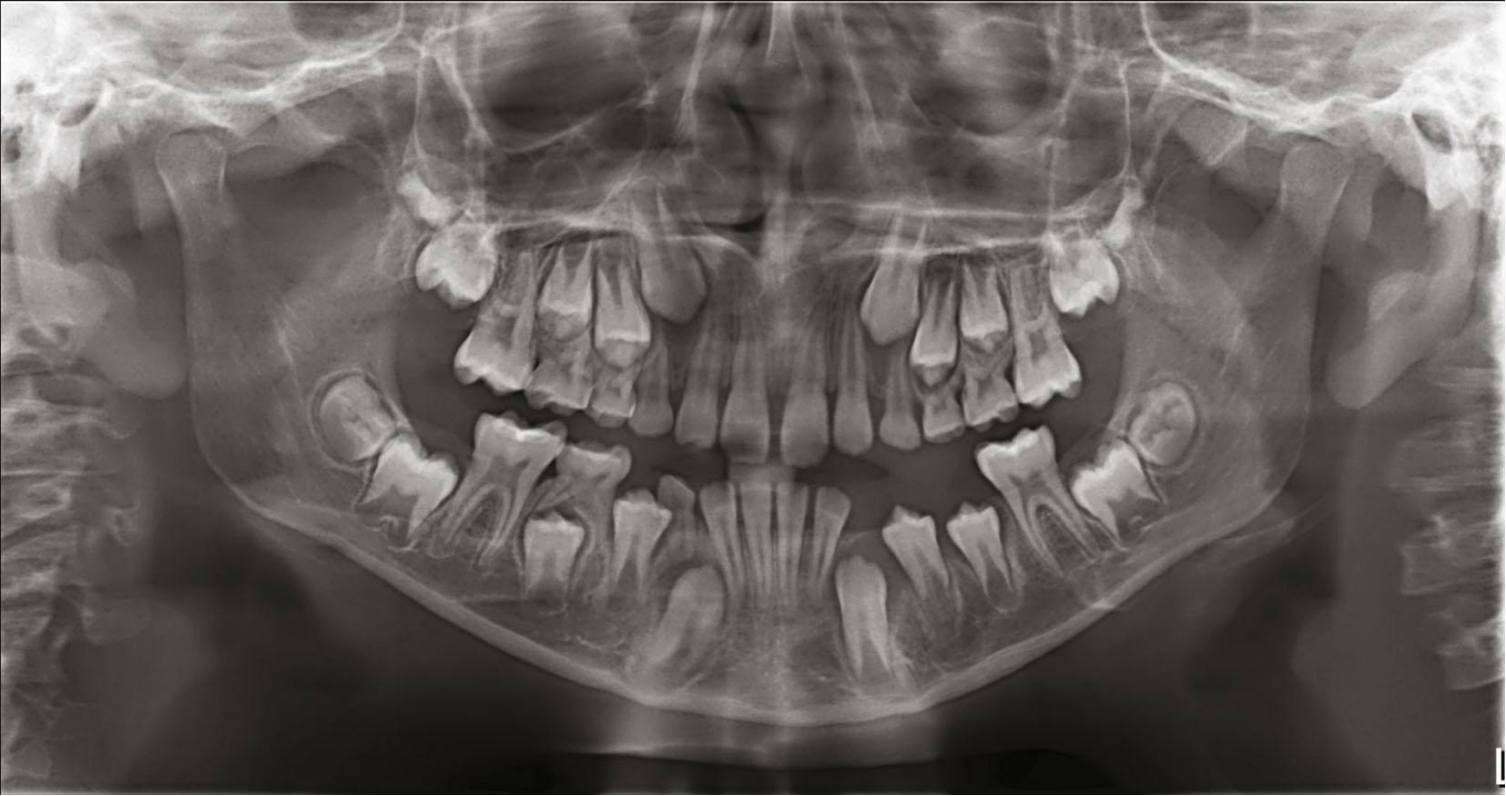
One-year panoramic radiographic follow-up showed the remarkable eruptive progression of the permanent teeth.

**Figure 8:**
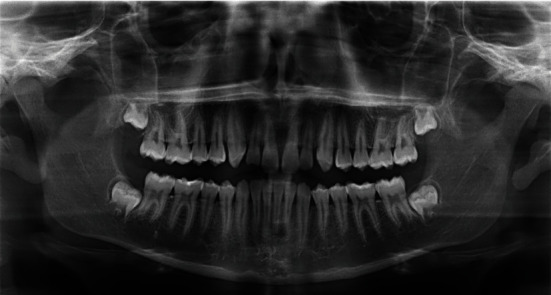
Five years later, complete bone regeneration, and positioning of teeth #33, #34, and #35 within the arch are reported with no evidence of recurrence.

The extraction of teeth #73, #74, and #75 was planned associated with the lesion decompression by inserting a drain in the extracted sockets area, followed by the placement of a space maintainer. The teeth were extracted under local anesthesia and curettage as an incisional biopsy of the cystic tissue was performed for histopathological analysis (Figures 3(a) and 3(b)). A sterile silicon irrigation tube used in implant surgery was cut into a small cylinder to serve as a drain. The tube was stabilized in the extracted sockets with multiple interrupted 3.0 nylon sutures (Figures 3(c) and 3(d)) and trimmed in length to avoid occlusal interference. Parents were instructed about rinsing the fixed system with saline and 0.12% chlorhexidine solutions, respectively, once and twice daily. Medical prescription included amoxicillin 500 mg twice daily for one week along with paracetamol 500 mg for pain management. Weekly recall examinations were scheduled. The histopathological results reported the characteristics of a dentigerous cyst (Figure 4). After one month of decompression, the fixed device was removed (Figure 5), and the patient was referred to his pedodontics' to have a space maintainer placed. At the six-month recall examination, the clinical (Figure 6(a)) and radiographic findings (Figure 6(b)) revealed bone regeneration in the previously cystic region, the eruption of the first permanent premolar, and the progression of the permanent canine and second premolar teeth buds towards occlusion. One-year radiographic follow-up showed the remarkable eruptive progression of the permanent teeth (Figure 7). Five years later, complete bone regeneration, and good positioning of teeth #33, #34, and #35 within the arch are reported with no evidence of recurrence (Figure 8).

## 3. Discussion

A cystic lesion can be non-odontogenic or odontogenic, the latter being inflammatory or developmental [10]. The dentigerous or follicular cyst belongs to the developmental category and is usually related to an impacted tooth. It is most frequently related to the mandibular third molar but its association with deciduous teeth has also been reported [11] with a slight prevalence in male patients [12]. The pathogenesis of those cysts has been described as the accumulation of exudate between the enamel epithelium and enamel or between the enamel organ's layers. This process is considered to be caused by the pressure applied by the erupting tooth on the follicle, resulting in venous constriction [3]. Studies show that cystic enlargement is due to a rise in the intraluminal osmotic pressure associated with continuous hydrostatic pressure on the peripheral bone [8]. Having an asymptomatic feature, dentigerous cysts get silently large enough to cause bone expansion and swelling until they get noticed, which was observed in this case. The treatment of this type of cyst in the mixed dentition depends on the size and location of the lesion, its epithelial lining status, and its proximity to vital structures, such as the adjacent teeth, the inferior alveolar nerve, and the mental foramen [4, 13]. The advantages of this technique are to reduce the pressure inside the cystic cavity, which will allow a better bone apposition. The involvement of anatomical structures and the bone plate status is better assessed with CT scans when the cyst is extensive as presented in our different scan cuts. However, diagnosis cannot be confirmed without histopathological reports to eliminate the possibility of more aggressive lesions that require additional or different interventions. An incisional biopsy of the cystic tissue sent to histopathological analysis confirmed the dentigerous feature of the lesion. Several treatment modalities are proposed for the management of dentigerous cysts, namely enucleation, marsupialization, and decompression. Enucleation consists of complete surgical removal of the cyst without rupture of its lining, and it is indicated for small-sized cysts (less than 5 cm) [4]. With larger lesions, this technique can result in mandibular fracture, devitalization of adjacent teeth, and damage to the permanent teeth germs, hence the consideration of conservative approaches such as marsupialization and decompression [2, 13]. Marsupialization was described by Partsch in 1892 and is achieved by deroofing the cyst through a large window within the bone and suturing the edges of the incised mucosa to the cystic wall to make it continuous with the oral cavity [6, 14]. Stanbouly et al. [9] suggested that intraluminal pressure can be decreased by a reduced opening in the cystic wall only to allow drainage of the lesion's content and enable secondary bone healing and formation. Decompression helps to maintain communication between the cyst and the oral medium through a fixed device, such as an acrylic stent or a disposable tube [7, 8, 15] similar to the trimmed irrigation tube that we used as a drain in this report. As per Masuda et al. [16], it is recommended to avoid enucleation if the cyst is in close contact with developing germs, and in our case, the unerupted teeth were involved with the cystic lesion. In addition to the expected surgical damages, our patient was too young to undertake cystectomy as it would have had a direct functional, esthetic, and psychological impact on him. Therefore, we planned a more conservative approach that was approved by the parents, and which was to decompress the lesion. This technique allowed the preservation of oral tissues and pulp vitality of adjacent teeth while avoiding the risk of unerupted teeth damage [17]. The affected primary teeth, however, had to be extracted for severe root resorption and to allow the fixing of the drainage tube. The eruptive potential of the cyst-associated teeth #34 and #35 was reported by their progressed root formation and open apices. It was an indicator of the optimal timing for intervention; and other criteria on which treatment selection was based [18, 19]. A space maintainer was placed to prevent teeth drifting that would interfere with the eruption of the permanent successors and to avoid orthodontic treatments in the future. It is important to note that the decompression technique is a patient-sensitive treatment protocol, which is its major drawback. Patient commitment is required; well-maintained hygiene and irrigation are essential due to the high risks of tube obliteration or infection [20]. Regular recall exams are scheduled to monitor healing and are usually extended over a long period of time. They ideally last till the complete eruption of affected teeth and bone ossification. Our patient was followed up for five years, the radiographic results of this case show no evidence of recurrence and suggest that the decompression of large dentigerous cysts is a reliable treatment modality in children even with severe displacement of teeth.

## 4. Conclusion

Children have higher bone regeneration capacity than adults and developing teeth with open apices have a remarkable eruptive potential which encourages treating large dentigerous cysts in the mixed dentition with the decompression technique. This case report highlights the importance of adapting the appropriate treatment protocol to the ideal intervention time. However, patient cooperation has a major role in determining the treatment's outcome noting that conservative approaches are prolonged therapies that need periodic evaluation and a commitment to specific hygienic measures.

## Data Availability

All available data are in the manuscript.
